# Effect of Chirality and Amphiphilicity on the Antimicrobial Activity of Tripodal Lysine-Based Peptides

**DOI:** 10.1021/acsabm.4c01635

**Published:** 2025-01-10

**Authors:** Anindyasundar Adak, Valeria Castelletto, Lucas de Mello, Bruno Mendes, Glyn Barrett, Jani Seitsonen, Ian W. Hamley

**Affiliations:** † School of Chemistry, Pharmacy and Food Biosciences, University of Reading, Whiteknights, Reading RG6 6AD, U.K.; ‡ School of Biological Sciences, University of Reading, Reading RG6 6AS, U.K.; § Nanomicroscopy Center, Aalto University, FIN-02150 Espoo, Finland

**Keywords:** peptides, tripodal, trifunctional, self-assembly, cytocompatibility, hemocompatibility, antimicrobial

## Abstract

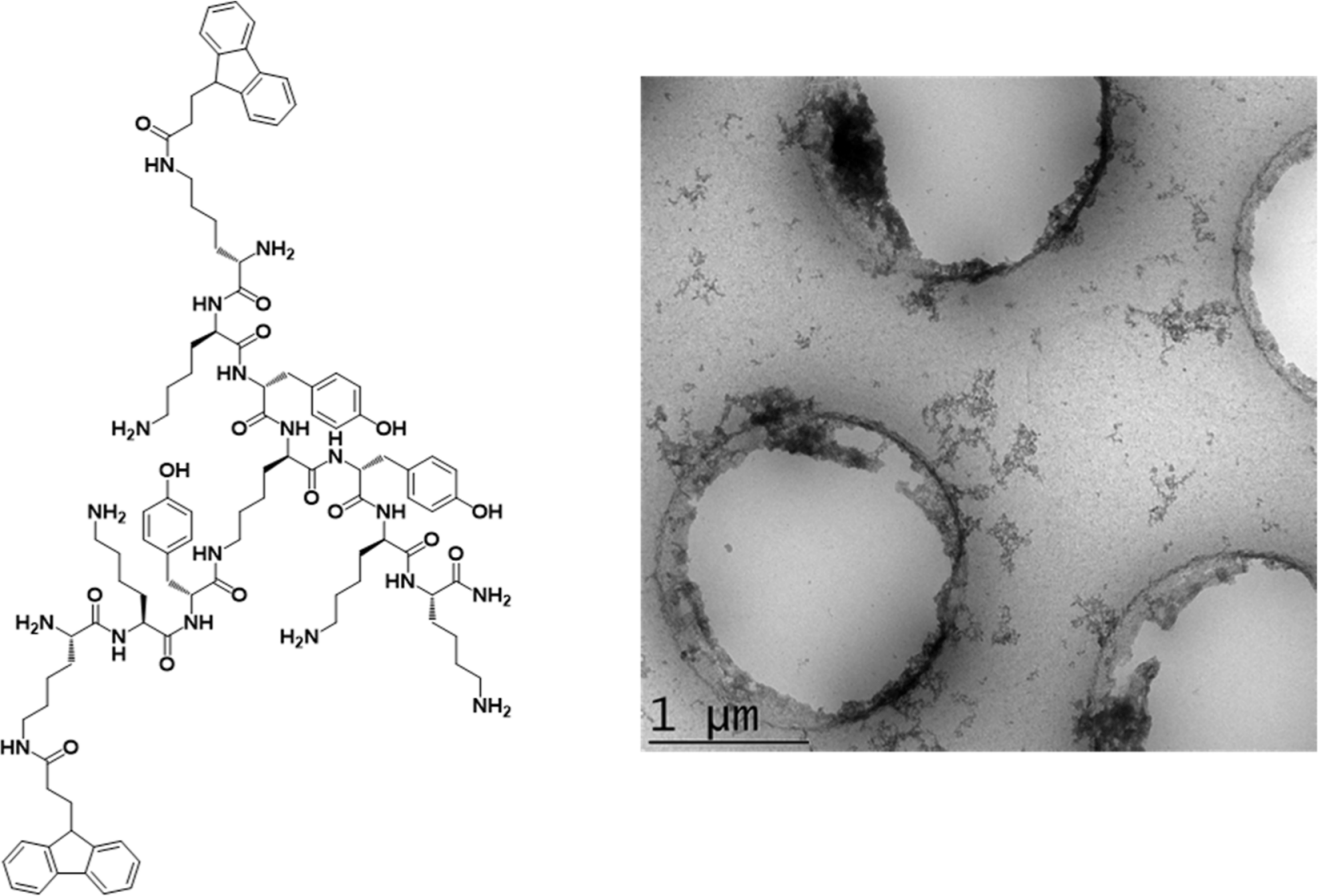

A series of tripodal (three-arm) lysine-based peptides were designed and synthesized and their self-assembly properties in aqueous solution and antimicrobial activity were investigated. We compare the behaviors of homochiral tripodal peptides (KKY)_3_K and a homologue containing the bulky aromatic fluorenylmethoxycarbonyl (Fmoc) group Fmoc-(KKY)_3_K, and heterochiral analogues containing k (d-Lys), (kkY)_3_K and Fmoc-(kkY)_3_K. The molecular conformation and self-assembly in aqueous solutions were probed using various spectroscopic techniques, along with small-angle X-ray scattering (SAXS) and cryogenic-transmission electron microscopy (cryo-TEM). In cell viability assays using fibroblast cell lines, the tripodal peptides without Fmoc were observed to be noncytotoxic over the concentration range studied, and the Fmoc functionalized tripodal peptides were only cytotoxic at the highest concentrations (above the critical aggregation concentration of the lipopeptides). The molecules also show good hemocompatibility at sufficiently low concentration, and antimicrobial activity was assessed via MIC (minimum inhibitory concentration) and MBC (minimum bactericidal concentration) assays. These revealed that the Fmoc-functionalized tripodal peptides had significant activity against both Gram-negative and Gram-positive bacteria, and in the case of Gram-positive *Staphylococcus aureus*, the antimicrobial activity for Fmoc-(kkY)_3_K was improved compared to polymyxin B. The mechanism of the antimicrobial assay was found to involve rupture of the bacterial membrane as evident from fluorescence microscopy live/dead cell assays, and scanning electron microscopy images.

## Introduction

The development of antimicrobial-resistance in bacteria is becoming an urgent healthcare challenge, and traditional antibiotic drugs are beginning to fail in response to emerging resistance.^1,2^ This has stimulated research on new types of antibiotics including antimicrobial peptides (AMPs), of which there are large numbers of native types from many organisms and which can also be created by de novo design of peptide sequences. Natural AMPs generally consist of 10–40 amino acids, which contain a net positive charge and a considerable number of hydrophobic residues. This amphiphilic character often facilitates their folding into a helix after binding to bacterial membranes.^3,4^ In multicellular organisms, AMPs are a key part of the innate immune system, which plays a crucial role as a defense shield against invading foreign pathogens. The antimicrobial property of AMPs is based on the ability of AMPs to disrupt bacterial membranes, leading to bacterial death.^5,6^ Researchers have identified over 4000 natural AMPs, as documented in several databases.^7−10^ Among them, cathelicidins, defensins, and histatins are human-specific AMPs, which play a critical role in the immune defense system.^11,12^

Inspired by natural AMPs, researchers worldwide have designed and developed various synthetic AMPs for effective antimicrobial activity. There are various classes of AMPs such as surfactant-like peptides (SLPs), which consist of single or multiple cationic residues forming the SLP headgroup, with a hydrophobic sequence (for example, oligoalanine) as a tail group.^13,14^ Due to their unique balance of amphiphilic character, they display various kinds of self-assembled nanostructures, such as fibrils, nanosheets, nanotapes, vesicles, and nanotubes.^15−17^ They also show significant antibacterial activity against common pathogens such as Gram-negative *Escherichia coli* and Gram-positive *Staphylococcus aureus* etc.^18−21^

Lipopeptides have also been created that show antimicrobial activity against microbial pathogens. Lipopeptides consist of a hydrophilic peptide (cyclic or linear form) and a hydrophobic lipid chain, and those for application as antimicrobials typically contain cationic residues lysine or arginine.^22−24^ Several key factors, modulate the antimicrobial activity of lipopeptides such as the peptide sequence, length of lipid chain, and the amphiphilicity of the lipopeptides. The lipopeptides generally disrupt the membrane of bacteria, and it has been proposed that this nonspecific mode of activity can reduce the emergence of microbial resistance. Lipopeptides can self-assemble into various nanostructures^25,26^ such as micelles,^27,28^ fibers,^29,30^ nanotubes,^31,32^ vesicles,^33^ nanosheets,^22,34^ nanotapes,^35,36^ and nanoribbons.^37,38^ There is great interest in the potential correlation of lipopeptide self-assembly and antimicrobial activity, and the interaction of lipopeptides with the bacterial membrane can cause pore formation in the lipid bilayer. The formation of self-assembled nanostructures of AMPs plays a crucial role in generating high local concentrations of the active peptide sequence to kill bacteria.^39,40^ Thus, self-assembling AMPs are promising candidates for antimicrobial applications. However, due to the low proteolytic stability of AMPs, cytotoxicity, and poor bioavailability, many clinical trials have been unsuccessful.^41,42^

To tackle these drawbacks, researchers focused more on designing and mimicking AMPs inspired by natural AMPs while keeping the essential properties for antimicrobial activity intact. In the design of AMPs, positively charged residues are installed to facilitate electrostatic interaction between these residues and negatively charged bacterial membranes. In contrast, hydrophobic residues are introduced to interact with the hydrophobic bacterial membrane core. Over the years, efforts have been made to understand the correlation between amphiphilicity and antimicrobial activity, for example, Porter et al. observed that amphiphilicity is a key factor for antimicrobial activity.^43^ The Boman research group also observed that increasing amphiphilicity resulted in better antimicrobial activity and lower toxicity.^44^ However, it was also noted that increased amphiphilicity resulted in increased toxicity and hemolytic activity.

Antimicrobial peptides (AMPs) are an important part of the host defense response to microbial infections and designed AMPs are also the subject of intense research activity to develop new approaches to overcome antimicrobial resistance. One key mechanism of antimicrobial activity of AMPs arises from their interaction with, and disruption of, bacterial membranes.^45,46^ The innate immune systems of both animals and plants involves AMPs, with a key role in host defense mechanisms.^47^ There is great interest in de novo designed or bioinspired AMPs containing hydrophobic regions which interact with the hydrophobic domain of the membrane, while cationic residues interact with the anionic outer parts of the bacterial cell wall, leading to disruption of the microbial membrane.^48,49^ Tryptophan, present in several major classes of native AMPs, has been identified as a hydrophobic aromatic residue with a particular preference for the interfacial region of lipid membranes.^50^ Lipidated peptides (lipopeptides) also show exhibit antimicrobial properties since the lipid chain can interact with the bacterial membrane, in addition to the activity of the peptide sequence.^51,52^ Lysine-rich lipopeptides comprising amyloid peptide fragments show promising antimicrobial and wound-healing properties.^24^ Lysine-based lipopeptides with homochiral or heterochiral sequences show antimicrobial activity.^53^ The self-assembly and antimicrobial activity of three lysine-based surfactant-like peptides, A_3_K, A_6_K and A_9_K have been examined.^54^ The antimicrobial properties were found to be dependent on the length of the hydrophobic alanine chain. The highly selective antibacterial activity of SLPs such as A_9_K_2_ has been demonstrated against both Gram-negative and -positive bacteria.^55^

The self-assembly and binding interaction between lipopeptides bearing short lysine-rich sequences and membranes has been studied by experimental and simulation methods, and both electrostatic and hydrophobic interactions were found to have important roles in the interaction with lipid bilayers.^56^ Cationic lipopeptides have been created that contain tryptophan and lysine residues, and significant antimicrobial activity was noted against multidrug-resistant pathogenic bacteria and fungi.^51^ A lipopeptide-based hydrogel containing a lipopeptide with a C_12_ lipid chain and (IIKK)_2_ peptide was shown to have significant activity in treating *Helicobacter pylori* infection.^57^ In related work, a hydrogel containing a double network structure of polymer and C_16_-WIIIKKK lipopeptide inhibited bacterial growth by sustained release of the lipopeptide.^58^ As well as lysine, arginine-based peptides and lipopeptides show significant promise in the development of AMPs due to the interaction of this residue with anionic membrane components.^16,19,50,59−63^

We previously reported antimicrobial homochiral and heterochiral lipopeptides, which not only showed various self-assembled nanostructures with the alternation of pH and chirality but also can kill bacterial pathogens effectively.^23,28^ To improve the antimicrobial activity we wanted to extend this work and design a more effective new type of peptide molecular structure. Tripodal peptides have been synthesized and show properties such as nanosphere formation from β-sheet forming sequences,^64^ vesicle (nanocage) formation from triskelions with aromatic dipeptide arms,^65^ pH-dependent self-assembly,^66,67^ or nanotorus formation from a biotinylated triskelion dipeptide.^68^ Applications include use as mimics of biologically active ligands with *C*_3_ symmetry^69^ or the creation of peptide-based nanosponges for cell-based cancer therapy,^70,71^ and one report demonstrated antimicrobial activity.^72^ Here, to understand the importance of amphiphilicity in antimicrobial activity, we designed a library of four tripodal (three-arm) homo and hetero chiral peptides.

The tripodal structure in our peptides enhances the presentation of cationic residues, with the aim to improve antimicrobial activity. The multivalent lysine-based peptides studied here are expected to have enhanced antibacterial properties. In addition, two derivatives containing Fmoc [fluorenylmethyloxycarbonyl] were prepared, this being a protecting group in peptide synthesis which may be left attached to peptides to drive self-assembly through π–π stacking interactions and hydrophobicity influencing amphiphilicity.^73^ The four molecules prepared are (i) (KKY)_3_K abbreviated as **TP**, (ii) Analogue (kkY)_3_K containing k (d-Lys) abbreviated as **DTP**, (iii) Fmoc-(KKY)_3_K abbreviated as **FTP** and (iv) its d-lysine analogue Fmoc-(kkY)_3_K abbreviated as **FDTP**. We have studied their conformation and self-assembled structure by CD, FTIR, Cryo-TEM, SAXS, and determined critical aggregation concentration (CAC) values. The cytocompatibility of the peptides was analyzed via cell viability and hemolysis assays and antimicrobial assays were performed to understand the effect of chirality and amphiphilicity on antimicrobial activity. A lead candidate molecule (**FTDP**) with excellent activity against Gram-negative species and Gram-positive *S. aureus* (enhanced compared to the widely used cyclic cationic lipopeptide polymyxin B) is identified.

## Experimental (Materials and Methods)

### Materials: Chemicals

Rink amide resin, Fmoc amino acids, diisopropylethylamine (DIPEA), and *O*-(1-benzotriazolyl)-1,1,3,3-tetramethyluronium hexafluorophosphate (HBTU), triisopropylsilane (TIS) were obtained from Sigma-Aldrich. Methanol, trifluoroacetic acid (TFA), piperidine, diethyl ether, phenol, dichloromethane, and *N*,*N*′-dimethylformamide (DMF), HPLC grade water, and HPLC grade acetonitrile were purchased from Thermo-Fisher. Lipopeptides were purified using an Agilent 1200 HPLC with a Supelco C-18 column (Zorbax ODS HPLC Column 15 × 4.6 mm, 5 μm) using a gradient of acetonitrile/water at a flow rate of 1.2 min/mL, for 30 min.

### Synthesis of Lipopeptides

The synthesis of the three arm trifunctional peptides was carried out following the solid phase peptide synthesis (SPPS) method. For the solid support, a resin (Rink amide) was used. For amide coupling between amino acids [Fmoc-amino acids (5 equiv)], the resin was purged by nitrogen gas for 6 h with a coupling mixture of HBTU (5 equiv), and *N*,*N*-diisopropylethylamine (DIPEA) (12 equiv)] in DMF. Similarly, the Fmoc group of each amino acid was deprotected by nitrogen purging for 20 min with (20% v/v) piperidine in DMF. After successful synthesis, the peptide was cleaved from the resin using a solution containing trifluoroacetic acid (TFA, 96%), triisopropylsilane (TIS, 2%), and H_2_O (2%). The cleavage was carried out for 4 h at 25 °C. After filtering the solution from the resin, excess TFA was removed by nitrogen gas. Next, the peptide was precipitated by adding cold ether. The solid peptide was obtained by performing centrifugation, lyophilization, and purification (HPLC, Agilent 1200 series). To examine the successful synthesis of the final molecule, the purity of the lipopeptides analyzed by HPLC are as follows: **TP** = 100%, **FTP** = 99.90%, **DTP** = 98.88%, **FDTP** = 99.86% (Figures S1, S3, S5, and S7). Electrospray ionization-mass spectrometry was also carried out (Figures S2, S4, S6, and S8). The observed mass matches with the expected exact molar masses for **TP** and **DTP**: *M*_theo_ = 1403.78 g mol^–1^, for **FTP** and **DFTP**: *M*_theo_ = 1848.27 g mol^–1^. The observed values are **TP**: *M*_obs_ = 1403.87 g mol^–1^, **FTP**: *M*_obs_ = 1848.01 g mol^–1^, **DTP**: *M*_obs_ = 1403.86 g mol^–1^, **FDTP**: *M*_obs_ = 1848.01 g mol^–1^.

### Sample Preparation

To prepare the samples, a weighed amount of solid powder of peptide was dissolved in ultrapure water to obtain the respective concentration (in wt %). The pH of these aqueous solutions was found to be stable at pH 6.5.

### CD Spectroscopy

The circular dichroism (CD) spectra of the peptides were obtained as described previously.^35^

### Fourier Transform Infrared (FTIR) Spectroscopy

The FTIR spectra of the lipopeptides were recorded as described previously.^35^

### Fluorescence Spectroscopy

Fluorescence experiments were carried out as described previously.^35^

To determine the CAC value of individual peptides, the results were plotted as *I*/*I*_0_ versus log(*c*/wt %), where *I* indicates the maximum fluorescence intensity of ANS at a given sample concentration and *I*_0_ denotes the peak intensity for the ANS solution without peptide.

### Cryogenic-Transmission Electron Microscopy (Cryo-TEM)

Imaging was carried out as described previously.^35,75^

### Small-Angle X-ray Scattering

SAXS experiments were performed on beamline SWING^76^ at synchrotron SOLEIL (Gif-sur-Yvette, France). The sample solutions were loaded into the 104-well plate of a custom built BioSAXS robot^76,77^ and then delivered to a quartz capillary in an evacuated chamber in the beam path. The sample-to-detector distance was 3436 mm with X-rays with energy 12.0 keV, i.e. wavelength λ = 1.033 Å. The images were captured using a Eiger X4M detector. Data processing (masking, radial averaging, background subtraction) was performed using dedicated beamline software FoxTrot. For each data set, 36 frames (0.99 s duration with 10 ms gap between frames) were acquired, with the sample under flow. Anomalous frames (resulting from insufficient sample injected in the beam etc.) were not included in the background subtraction.

### Cell Lines

L929 murine fibroblast cells (ECACC General Cell Collection) were grown in Dulbecco’s modified Eagle’s medium (DMEM) supplemented with 10% fetal bovine serum (FBS), 20 mM HEPES, and 1% GlutaMAX. The cells were maintained at pH 7.4, 37 °C, and 5% CO_2_ in 25 cm^2^ cell culture flasks.

### Cytocompatibility Assays

Assays were conducted as described previously.^23^

### In Vitro Evaluation of the Antibacterial Properties of Synthetic Peptides

The antibacterial activity of the peptides was evaluated on five bacterial strains: *E. coli* O157:H7 strain EDL933, *Salmonella enterica* serovar Typhimurium, *Klebsiella aerogenes*, *S. aureus* (ATCC 12600) and *Pseudomonas aeruginosa* (NCTC 13437), a clinical multidrug-resistant isolate. The killing activity of peptides on bacteria was examined based on the determination of two quantities: minimal inhibitory concentration (MIC) and minimal bactericidal concentration (MBC).^79^ Data was collected from three independent assays carried out in triplicate. For all strains, cultures from our frozen glycerol stocks were initially streaked onto Luria–Bertani (LB) agar plates. A single, isolated colony of each bacterium was grown in 5 mL LB broth in a shaking incubator (3 7 °C, 250 rpm). Aliquots 150 μL at a cell density of 5 × 10^6^ CFU mL^–1^ were transferred to 96-well U-shaped microplates following the International Organisation for Standardisation (ISO) 20,776-1 and exposed to a range of concentrations (0–1 mg mL^–1^) of tested peptides for 24 h. Polymyxin B (a lipopeptide-based antibiotic) and water were used as positive and negative controls, respectively. Bacterial growth was measured through absorbance readings on a Tecan Spark microplate reader at 600 nm. The MIC was defined as the lowest concentration, where no change in absorbance, relative to the negative control, was detected. MIC values were expressed as the mean from three independent experiments. MBC, the lowest concentration at which no viable colonies were observed, was determined by transferring 5 μL aliquots from the wells of the test microplate, starting with the wells corresponding to the MIC and those containing 2- and 4-fold higher concentrations of the MIC to Mueller–Hinton Agar (MHA) plates.

### Microscopic Analysis of Peptide-Induced Membrane Alterations

Dual staining of peptide-treated bacteria, to detect potential membrane disruption,^80^ was performed using a bacteria live/dead staining kit (PromoKine). This process involves incubating samples with fluorescent, nucleic acid dyes (DMAO, green, and EthD-III, red) that, respectively, are either able or unable to cross the bacterial membrane. Before staining, *E. coli* EDL933 cells were treated with peptides exhibiting antibacterial properties at their MBC for 1 h at 37 °C. Next, they were incubated with set volumes of both dyes for 15 min at room temperature. Slides were mounted using 5 μL of the stained solution onto glass coverslips and observed under a Nikon Eclipse Ti inverted microscope using a 10× objective. Images of different treatments, including controls were captured and compared.

### Scanning Electron Microscopy (SEM)

SEM imaging was performed on *E. coli* EDL933 cells processed under the same conditions described above. Cells were fixed with 2.5% glutaraldehyde for 18 h at 4 °C, then dehydrated using a graded ethanol series of 50%, 70%, 80%, 90%, 95%, and 100% for 15 min at each step. Finally, 5 μL of the suspension was placed onto coverslips and coated with gold. Images were obtained using a Cambridge Instruments Stereoscan 360 microscope.

### In Vitro Analysis of Cytotoxic Effects of Synthetic Peptides

Cytotoxicity was evaluated using human red blood cells (hRBCs) and murine fibroblasts (L929). In the first assay, erythrocytes were carefully isolated from donated human blood following centrifugation at 1000*g* (10 min; 4 °C). Fresh anucleate cells were washed three times using PBS and centrifuged at 700*g* (10 min; 4 °C). Then, a solution of washed hRBCs 0.5% (v/v) was prepared in PBS and aliquots were transferred to 96-well plates. A range of peptide concentrations (0–0.1 wt %.) were added to each well and incubated (1 h; RT). Cell leakage was monitored through hemoglobin detection at 414 nm using a Tecan Spark microplate reader. The hemolytic effect was normalized based on the absorbance readings of hRBCs incubated with 0.1% (v/v) Triton X-100 (TX-100). The toxicity to hRBCs was expressed as a percentage applying the equation
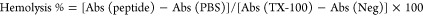
where: Abs (peptide) = absorbance of each sample well; Abs (Neg) = averaged absorbance of the negative controls (PBS), and Abs (TX-100) = averaged absorbance of the positive controls (Triton X-100). No release of hemoglobin was confirmed in the negative control, where PBS was added in place of peptides in the solution.

## Results and Discussion

**Figure 1:**
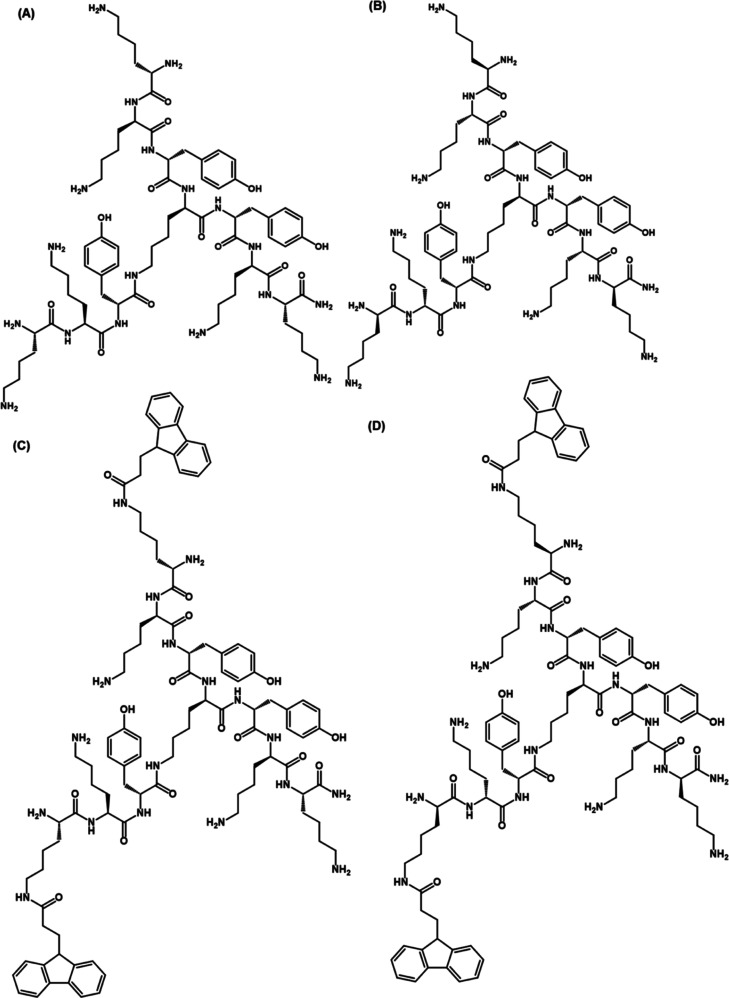
Structure of the library of trifunctional peptides: (A) **TP** with each lysine as l-isomer, (B) **DTP** with each lysine as d-isomer except the central lysine, (C) **FTP** with each lysine as l-isomer, and containing two Fmoc groups, and (D) **FDTP** with each lysine d-isomer except the central lysine, and containing two Fmoc groups.

As part of our program to develop lipopeptides with enhanced antimicrobial activity, we prepared peptides with enhanced presentation of cationic lysine residues. The structures of the four tripodal (three-arm) peptides synthesized are shown in Figure 1 and correspond to the following: (i) (KKY)_3_K abbreviated as **TP**, (ii) analogue (kkY)_3_K containing k (d-Lys) abbreviated as **DTP**, (iii) Fmoc(KKY)_3_K abbreviated as **FTP**, and (iv) analogue Fmoc-(kkY)_3_K containing k (d-Lys) abbreviated as **FDTP**. All molecules were synthesized by solid-phase peptide synthesis methods.

All the peptides were characterized by electrospray-ionization mass spectrometry (ESI-MS), and their purity was checked by reverse phase HPLC (RP-HPLC). The characterization data is shown in Figures S1–S8. The compounds have the expected molar masses and high purity (>98%).

**Figure 2:**
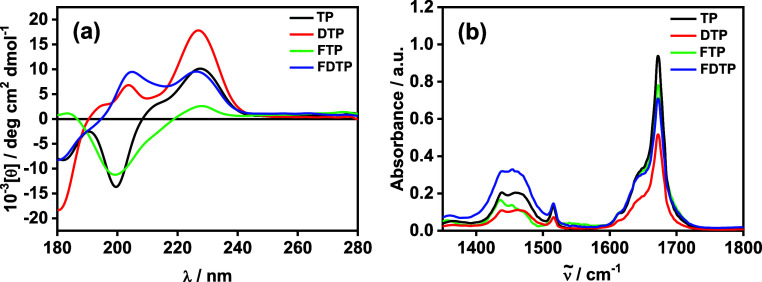
(a) CD spectra, (b) FTIR spectra from 0.5 wt % aqueous of the molecules as indicated.

The peptide conformation was probed using spectroscopic methods. Circular dichroism (CD) spectra are shown in Figure 2. In the CD spectra, the 0.5 wt % aqueous solutions of homochiral tripodal peptides **TP** and **FTP** displayed a minimum with a negative band at 200 nm followed by a positive band near 230 nm, which suggests unordered and/or extended (PPII, polyproline II-like) coil conformation (Figure 2a). The peak at 230 nm also contains a significant contribution from the aromatic tyrosine residues.^81−83^ The spectra for heterochiral tripodal peptides **DTP** and **FDTP** show a positive peak at 205 nm and a maximum near 230 nm which again suggests unordered and/or extended (PPII-like) coil conformation (Figure 2a). The presence of PPII-like conformations was tested using the denaturing solvent 6 M guanidine hydrochloride, and the spectra are shown Figure S9 and indeed an increase in the maximum near 230 nm is observed, as expected.^84^ This is more notable for some of the peptides, especially **FDTP** for which shifts in the position of the maximum are also evident. Spectra at additional concentrations are included in Figure S9.

FTIR spectra were measured to provide additional information on peptide conformation. Spectra covering the amide I and II regions are shown in Figure 2b. From Figure 2b, a broad shoulder at 1650 cm^–1^ was observed for the tripodal peptides (along with a peak at 1672 cm^–1^, which is due to TFA counterions bound to the peptide),^85^ which suggests of the presence of disordered conformation.^86−88^ For all the lipopeptides a broad peak in the amide II’ region centered around 1440–1460 cm^–1^ is evident, which is due to N-H/C-N deformation modes.^87^

**Figure 3:**
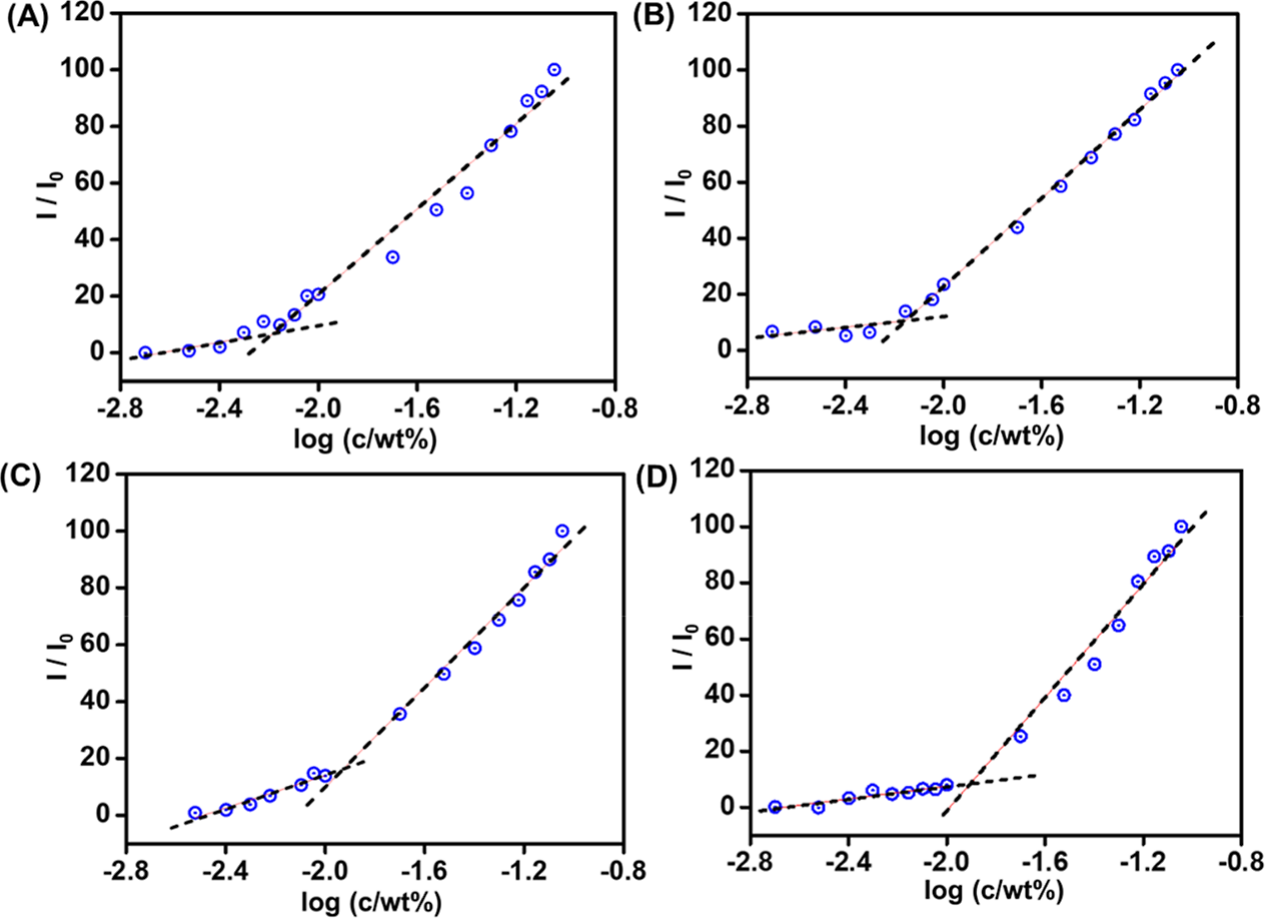
CAC assay using ANS fluorescence peak intensity to determine the CAC value for (A) **TP**, (B) **DTP**, (C) **FTP**, (D), and **FDTP**.

Critical aggregation concentration (CAC) values of the tripodal peptides were obtained from fluorescence probe assays using 8-anilo-1-naphthalenesulfonic acid (ANS). At higher concentrations, an emission peak at 486 nm is visible (original spectra shown in Figure S10). By plotting the fluorescence intensity (*I*/*I*_0_) at 486 nm as a function of the concentration (Figure 3), the concentration at the breakpoint (corresponding to the CAC) was found to be 0.0065 ± 0.004 wt % for **TP** (Figure 3A), 0.0069 ± 0.002 wt % for **DTP** (Figure 3B), 0.0113 ± 0.005 wt % for **FTP** (Figure 3C), 0.0123 ± 0.003 wt % for **FDTP** (Figure 3D).

**Figure 4:**
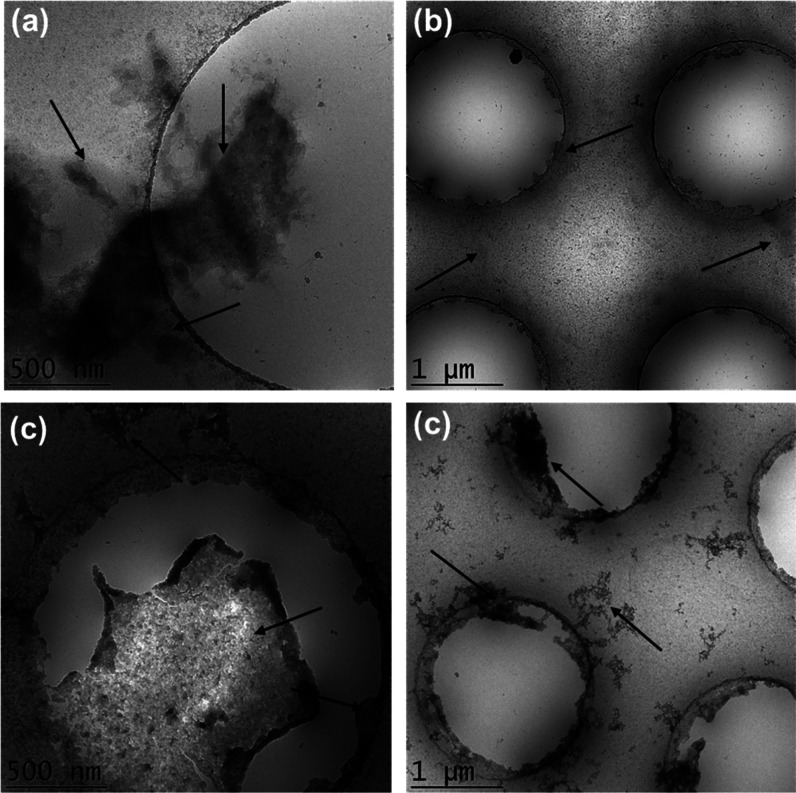
Cryo-TEM images from 1 wt % solutions of three arm peptides (a) **TP**, (b) **DTP**, (c) **FTP**, (d) **FDTP**. The arrows highlight selected nanostructures.

To examine the morphology of the self-assembled nanostructure of these three arm trifunctional peptides, cryo-TEM imaging was performed for 1 wt % solutions, above the measured CAC values. The images shown in Figure 4a,b,d show irregular globular aggregates for **TP**, **DTP** and **FDTP**. In many cases, these structures are deposited on the lacey carbon grid in preference to, or in addition to, the vitrified grid holes. This indicates that the aggregates have a hydrophobic character. In contrast to the other samples, for **FTP**, sheet-like structures were observed (Figure 4c).

**Figure 5:**
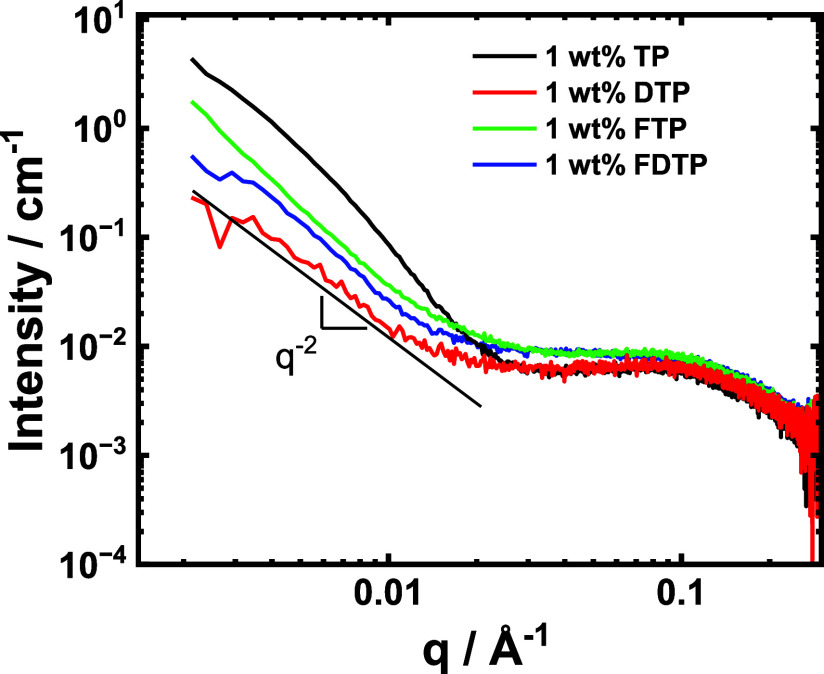
SAXS data for the four trifunctional tripodal peptides as indicated.

SAXS provides in situ structural information on aggregation, to complement cryo-TEM. SAXS data are presented in Figure 5 and the data for all four peptides shows an upturn in intensity at low wavenumber *q* that is a signature of aggregation, along with a plateau at high *q* that is due to monomer structures. The data are relatively featureless so that unique form factor fitting from well-defined structures are not possible (the lack of such structures is apparent from the cryo-TEM images). The limiting slope of the intensity at low *q* is *I*(*q*) ∼ *q*^–*n*^ with *n* = 1.5–2.2 which is consistent with planar structures, as observed in particular in the cryo-TEM images for **FTP**. Based on the magnitude of the forward (low *q*) scattering, samples **TP** and **FTP** show the most aggregated structure, which is consistent with the cryo-TEM images in Figure 4.

**Figure 6:**
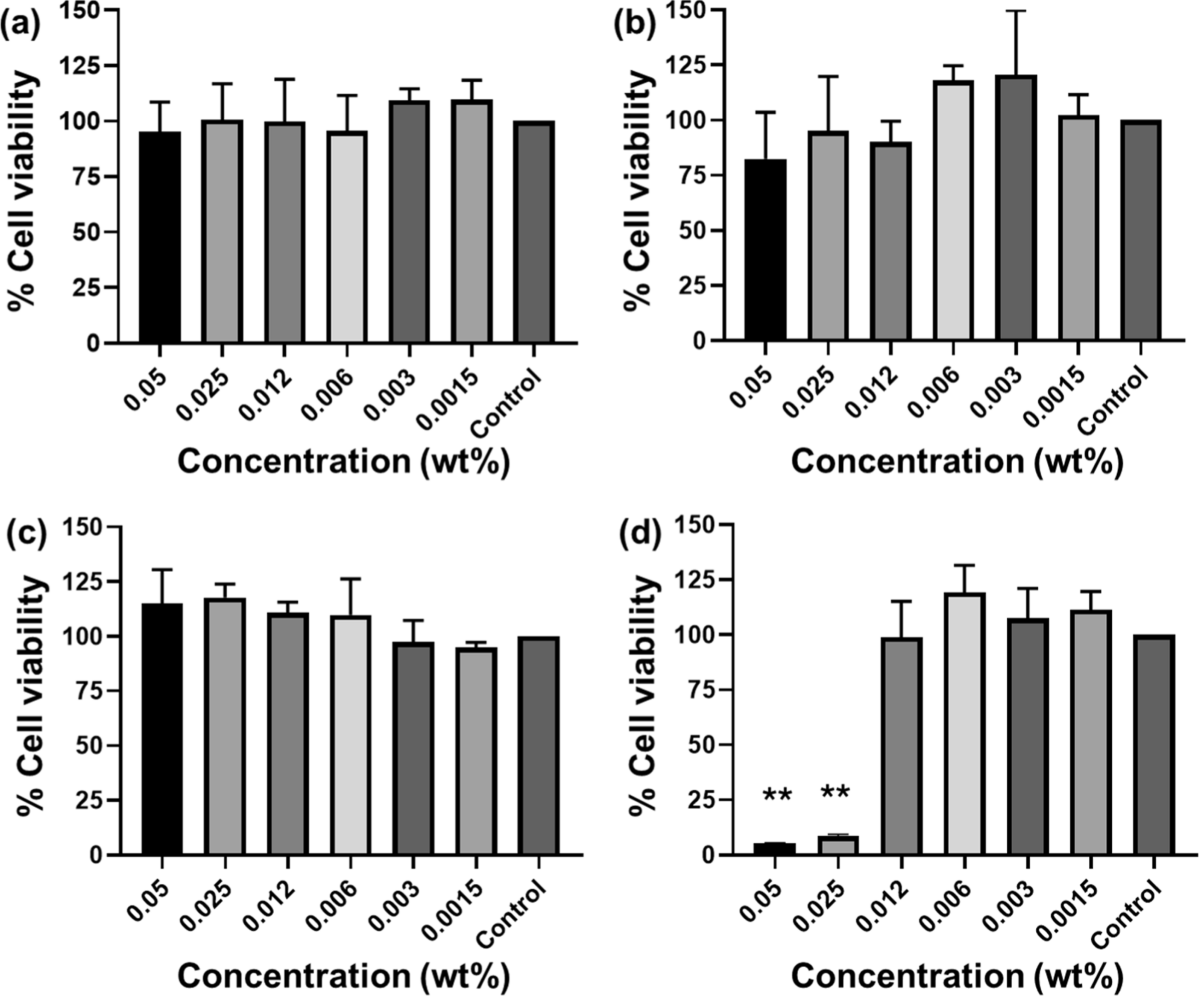
Cell viability data (with L929 murine fibroblasts) of tripodal peptides: (a) **TP**, (b) **DTP**, (c) **FTP**, and (d) **FDTP**. The error bar corresponds to the standard deviation of the value from the mean (*n* = 3, *p < 0.05, **p < 0.01 by performing the Anova statistical test).

To be of utility as practical antimicrobials, bioactive molecules must show selective activity compared to their toxicity toward mammalian cells. The cytocompatibility of our peptides was investigated by a cell metabolic activity assay using MTT [3-(4,5-dimethylthiazol-2-yl)-2,5-diphenyltetrazolium bromide] assays using L929 murine fibroblast cell lines. The data in Figure 6 show that the cell viability (after 48 h) is very high (there was no significant difference when compared to control cells) for all concentrations examined for the tripodal peptides except **FTDP** at the highest concentration (Figure 6), suggesting that the molecules are noncytotoxic in nature at sufficiently low concentration.

**Figure 7:**
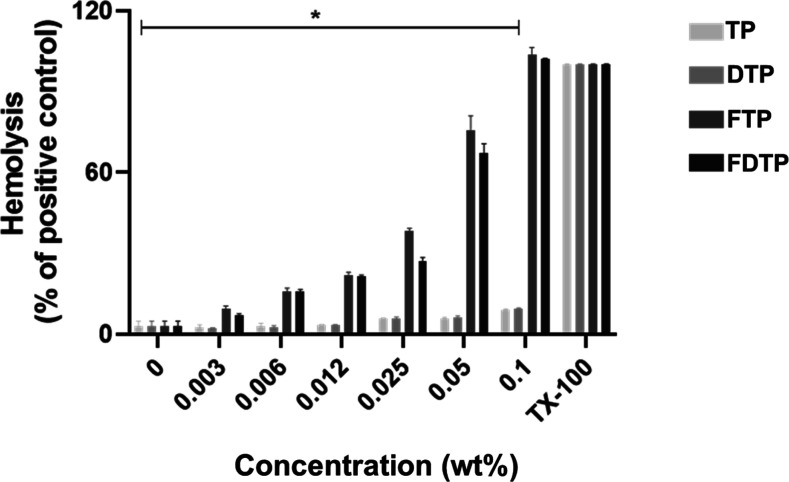
Hemolysis of human red blood cells (hRBCs) induced by various peptide concentrations. The hemolytic activity of tripodal peptides against hRBCs was assessed in a 96-well plate. hRBCs were exposed to a 2-fold serial dilution of the peptides and incubated for 1 h at 37 °C. Hemoglobin release, indicative of cell lysis, was measured using spectrophotometry at 414 nm and compared to a positive control (Triton X-100). Data were analyzed using one-way ANOVA followed by Tukey’s posthoc multiple comparison tests. Results are presented as the mean of three independent experiments, with the bar indicating statistically significant differences relative to the positive control (*P < 0.001).

In this study, we used two human cell types (fibroblasts and hRBCs) to assess peptide cytotoxicity. The hemolysis assay data is shown in Figure 7. This shows that **TP** and **DTP** did not lyse hRBCs (and nor do they or reduce the viability of fibroblasts, Figure 6). Fmoc-peptides, particularly **FDTP** resulted in, greater leakage of hRBCs (Figure 7) and death of fibroblasts (Figure 6). However, these toxic effects were notable at concentrations higher than both MIC and MBC (for instance from values for the Fmoc-peptides in Table 1, 62.5 μg/mL corresponds to 0.00625 wt %), indicating a potentially safe therapeutic window. In other words, the antimicrobial Fmoc-peptides characterized in this study have a selective nature, acting at lower concentrations in prokaryotic cells.

**Table 1:** Antibacterial Assay Showing MIC and MBC Values of the Three Arm Peptides against Both Gram-Negative and Gram-Positive Bacteria after 24 h Incubation Time

	MIC/MBC (μg/mL)				
	**TP**	**FTP**	**DTP**	**FDTP**	polymyxin B
Gram-Negative					
E. coli (O157:H7 strain EDL933)	>1000/>1000	62.5/125	500/1000	15.62/31.25	3.12/6.25
Pseudomonas aeruginosa (NCTC13437)	>1000/>1000	125/250	>1000/>1000	125/250	6.25/25
S. enterica (S. Typhimurium)	>1000/>1000	250/500	>1000/>1000	250/500	6.25/12.5
K. aerogenes	>1000/>1000	62.5/62.5	>1000/>1000	62.5/62.5	3.12/6.25
Gram-Positive					
S. aureus (ATCC 12600)	>1000/>1000	125/250	>1000/>1000	31.25/62.5	>100/>100
S. pyogenes					

Antimicrobial activity was quantified through determination of MIC and MBC values (Table 1) for all peptides, as well as bacterial live/dead assays to probe bacterial membrane integrity and SEM imaging of bacteria. The antibacterial studies revealed two promising chemical structures for further antibiotic development. While **TP** and **DTP** did not compromise the viability of Gram-positive or Gram-negative bacteria at low concentrations, both **FTP** and **FDTP** showed strong antibacterial activity (Table 1). Thus, Fmoc groups appear to enhance the antibacterial activity of peptides, making them more promising candidates for antibiotic development. The incorporation of this group has been used in previous studies to improve antimicrobial effects.^89−91^ The mechanism of antimicrobial action of Fmoc-containing peptides (including hydrogels) has been ascribed to a combination of the self-assembled β-sheet fibril structure (as for amyloid β, which has antimicrobial properties^92^), and the peptide hydrophobicity.^91^ Surprisingly, it has been reported that Fmoc-FF has better activity against microbial biofilms than Fmoc-FF with additional cationic residues (Fmoc-FFKK, Fmoc-FFFKK or Fmoc-FFOO, O: ornithine).^89^ In our work, **FDTP** demonstrated the lowest MIC and MBC values across all tested strains. Interestingly, **FDTP** exhibited greater activity against *S. aureus* compared to polymyxin B (Table 1), a peptide-based antibiotic used in clinical settings to treat infections due to Gram-negative bacteria, with limited efficacy against Gram-positive bacteria.^93^

**Figure 8:**
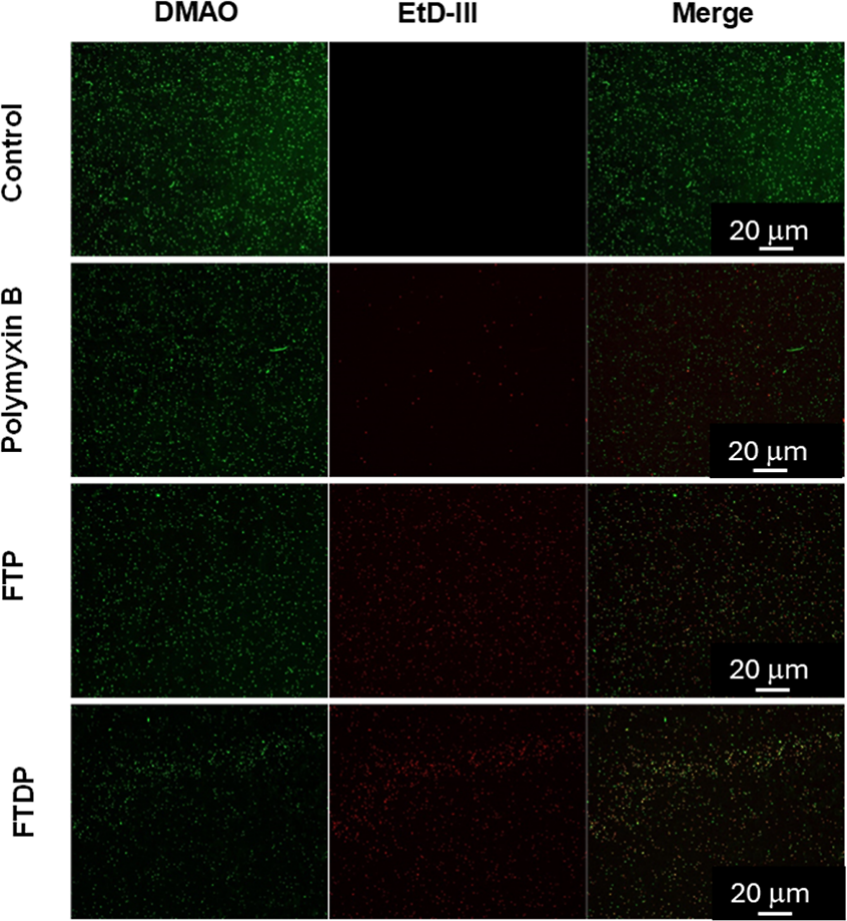
DMAO/EtD-III stained images showing the peptide effects on the integrity of the bacterial membrane. Fluorescence microscopy was used to visualize DMAO and EtD-III dyes binding to the bacterial DNA. In this experiment, *E. coli* EDL933 was incubated with peptides at their minimum bactericidal concentration (MBC) for 1 h at 37 °C, followed by staining with a bacteria live/dead staining kit (PromoKine). Cells with disrupted membranes are stained red (EtD-III), while cells with intact membranes are stained green (DMAO). These images confirm the rapid, membrane-targeting effects of **FTP** and **FDTP** peptides. A similar effect was observed with the reference peptide antibiotic, polymyxin B (as a positive control) but not the negative control (PBS), all stained green.

**Figure 9:**
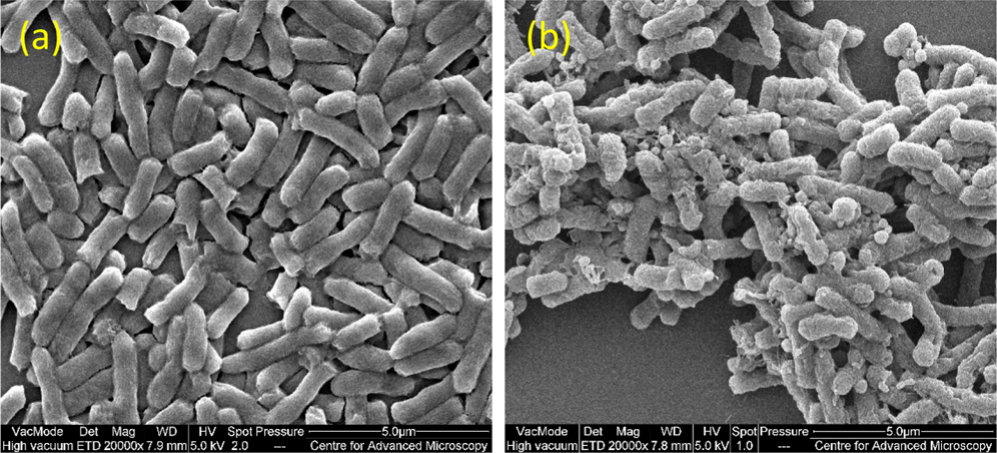
SEM images of *E. coli* EDL933 before (a) and after (b) incubation with **FTDP** at 31.25 μg/mL, showing normal rod-shaped bacteria and collapsed shapes, respectively.

Enhancing the bactericidal efficacy of novel antimicrobial peptides also requires a thorough understanding of their molecular, and cellular targets. Analysis of stained bacteria, using microscopy, indicated that active peptides (**FTP** and **FDTP**) act similarly to polymyxin B. Whereas live, control, cells fluoresce green, indicating an intact bacterial membrane architecture, bacteria treated with membrane-active antibiotics and antimicrobial peptides exhibited a high proportion of cells stained red, signifying compromised membrane integrity. The uptake of this dye suggests a membranolytic effect caused by the peptides, which likely induce changes in the membrane architecture, resulting in a loss of functionality and leakage of intracellular components. The Fmoc moiety in **FTP** and **FDTP** likely enhances their interaction with the hydrophobic core of phospholipid bilayers resulting in membrane disruption (Figure 8). This was further confirmed via SEM imaging of *E. coli* bacteria. Significant disruption of the surface of the bacteria was noted in the presence of **FDTP**, the outer membrane having a rough appearance with visible regions of damage (and extracellular debris visible), in contrast to the smooth and intact aspect of the native rod-like bacteria (Figure 9).

## Conclusions

In summary, we designed and synthesized a small library of tripodal lysine-based peptides, i.e. two homochiral tripodal peptides containing Lys: **TP** and **FTP** and two heterochiral tripodal peptides containing k (d-Lys): **DTP** and **FDTP**. The self-assembly of these tripodal peptides was examined in solution by cryo-TEM, and SAXS, which displayed globular aggregates, except for **FTP** which forms nanosheets. The CAC values of the molecules were determined from fluorescence probe measurements using ANS. The molecules have unordered conformations, as revealed by CD and FTIR spectroscopy. The cytotoxicity for all molecules at low concentrations from MTT assays using fibroblasts was low, although **FDTP** shows cytotoxicity at the two highest concentrations studied (above the critical aggregation concentration of the lipopeptides). All peptides are hemocompatible at low concentration although the two molecules containing Fmoc show significant hemolysis at higher concentration, although above the MIC and MBC values. In the antibacterial activity assays, the Fmoc tripodal peptides were more active than the analogues without Fmoc against both Gram-negative and Gram-positive bacteria. Peptide **FDTP** is our lead compound as it has the best activity against Gram-negative *E. coli* and Gram-positive *S. aureus*. The mechanism of the antimicrobial action was through disruption of the bacterial cell membrane as evident from the live/dead stained fluorescence images of bacteria, and SEM imaging. Our findings suggest that further chemical optimization of these peptide structures may be necessary to achieve lower MIC and MBC values. Amino acid substitution and/or lipidation strategies could be useful routes to re-engineer the structure and increase the efficacy of these agents. Alternative approaches could include coadministration in mixtures with other antibiotics, as this can provide synergistic effects.

## Supporting Information Available

Characterization data for peptides from HPLC and ESI-MS, concentration-dependent CD with and without guanidine hydrochloride, ANS fluorescence spectra (PDF)
