# Correction to “Effect of Chirality and Amphiphilicity on the Antimicrobial Activity of Tripodal Lysine-Based Peptides”

**DOI:** 10.1021/acsabm.5c00130

**Published:** 2025-02-07

**Authors:** Anindyasundar Adak, Valeria Castelletto, Lucas de Mello, Bruno Mendes, Glyn Barrett, Jani Seitsonen, Ian W. Hamley

In our original article, the molecular structures in Figure 1C and D missed an oxygen atom in the Fmoc groups. The corrected Figure 1 is shown below.

**Figure 1:**
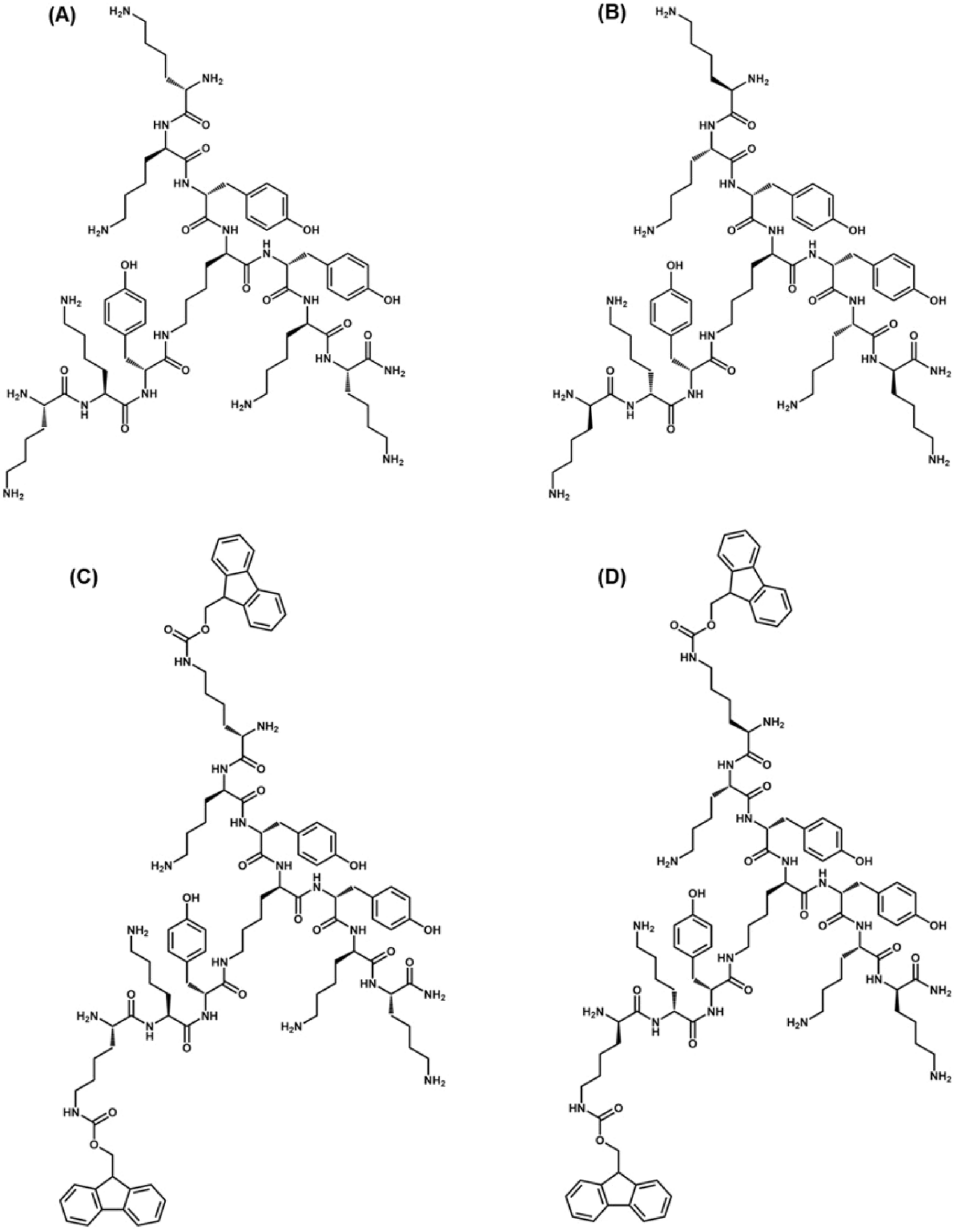
Structure of the library of trifunctional peptides: (A) **TP** with each lysine as l-isomer, (B) **DTP** with each lysine as d-isomer except the central lysine, (C) **FTP** with each lysine as l-isomer containing two Fmoc groups, and (D) **FDTP** with each lysine d-isomer except the central lysine, containing two Fmoc groups.

Similarly for the TOC entry, the correct version (for the structure of **FTP**) is shown below:
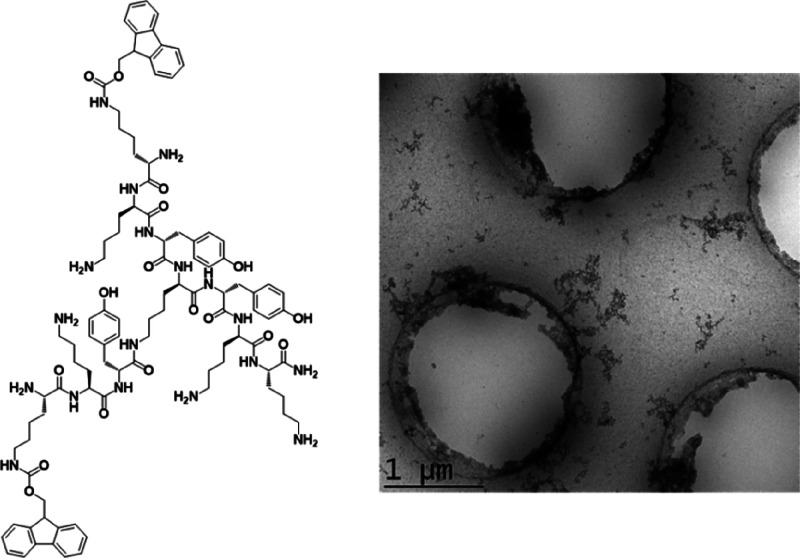


In addition, the CAC assay data presented in Figure 3 actually represent plots of (*I* – *I*_0_)/(*I*_max_ – *I*_0_), where *I* is the peak fluorescence at a given peptide concentration with ANS, *I*_0_ is that of the ANS solution, and *I*_max_ is the maximum ANS fluorescence intensity (for the highest peptide concentration). The corrected Figure 3 showing *I*/*I*_0_ is shown below.

**Figure 3:**
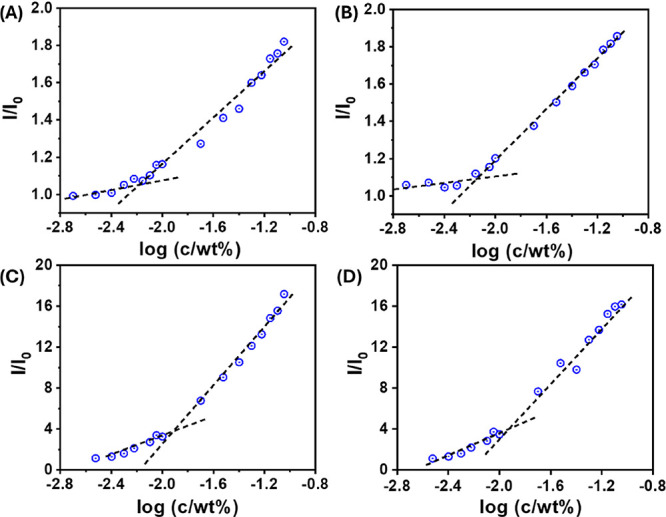
CAC assay using ANS fluorescence peak intensity to determine the CAC value for (A) **TP**, (B) **DTP**, (C) **FTP**, and (D) **FDTP**.

The values of CAC reported in the paper are unaffected. It may be noted that *I*/*I*_0_ reaches significantly higher values for the two peptides containing Fmoc due to the contribution to the fluorescence of this group.

