# Supplementary material for: Effect of Chirality and Amphiphilicity on the Antimicrobial Activity of Tripodal Lysine-Based Peptides
Source: ACS Appl Bio Mater. 2025 Jan 10;8(1):803–13. doi: 10.1021/acsabm.4c01635 (PMC11752523; doi:10.1021/acsabm.4c01635)
Supplement: Supplementary file 1 — mt4c01635_si_001.pdf [file mt4c01635_si_001.pdf]

Supporting Information

The Effect of Chirality and Amphiphilicity on the Antimicrobial Activity of Tripodal Lysine-Based Peptides

Anindyasundar Adak,[a] Valeria Castelletto,[a] Lucas de Mello,[a] Bruno Mendes,[b] Glyn Barrett,[b] Jani Seitsonen,[c] and Ian W. Hamley*[a]

[a] *School of Chemistry, Pharmacy and Food Biosciences, University of Reading, Whiteknights, Reading RG6 6AH, U.K.*

[b]*School of Biological Sciences, University of Reading, Reading RG6 6AH, U.K.*

[c]*Nanomicroscopy Center, Aalto University, FIN-02150 Espoo, Finland*

* I.W.Hamley@reading.ac.uk

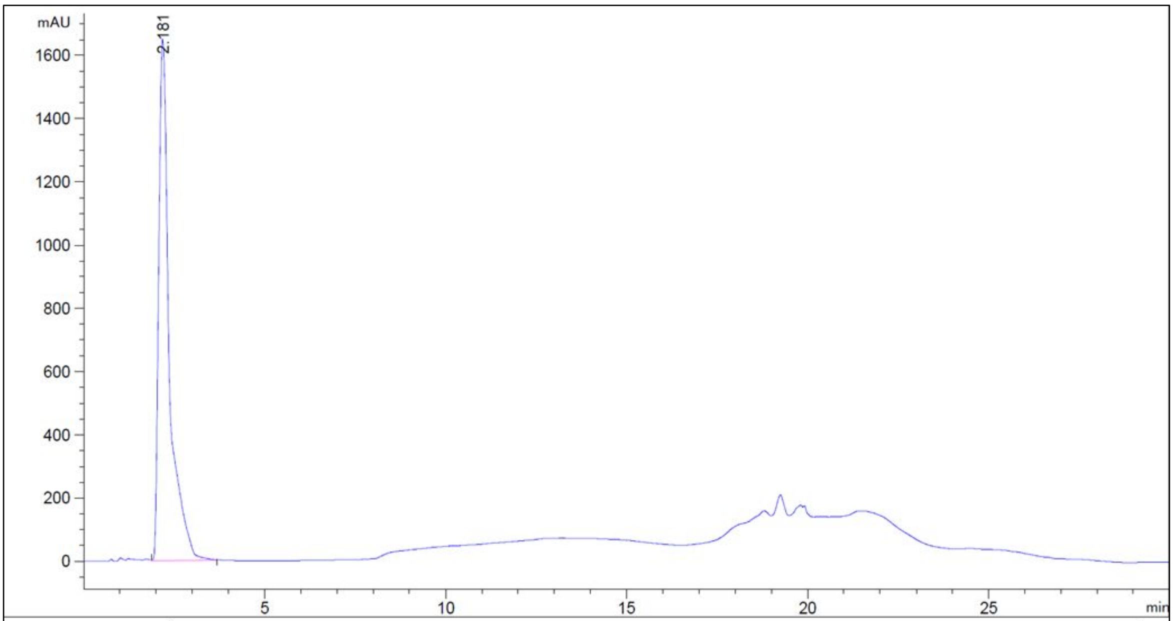

Peak #	RetTime [min]	Sig	Type	Area [mAU*s]	Height [mAU]	Area %
1	2.181	1	VB	3.39892e4	1648.94189	100.0000
Totals :				3.39892e4	1648.94189	

Figure S1. HPLC chromatogram of peptide **TP**.

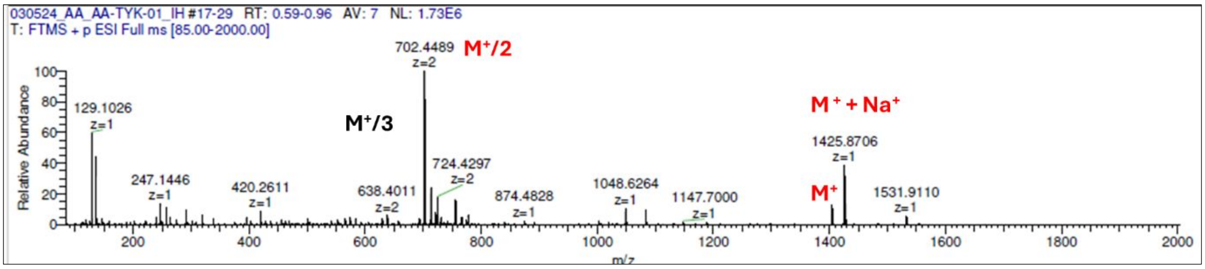

Figure S2. ESI-MS spectrum of peptide **TP**.

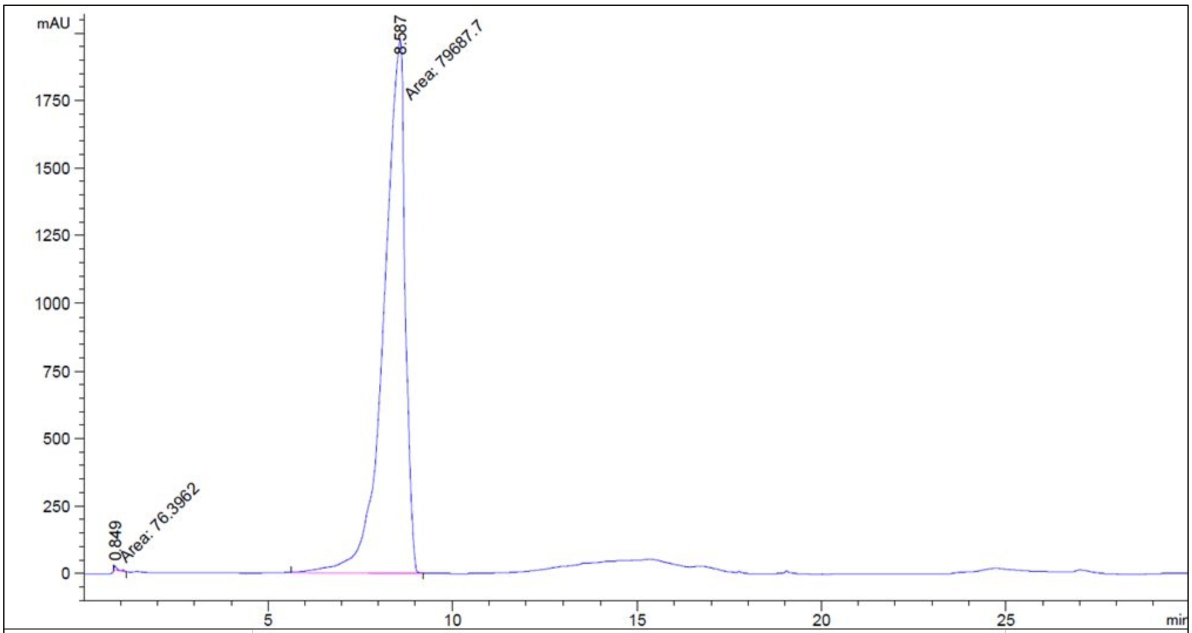

Peak #	RetTime [min]	Sig	Type	Area [mAU*s]	Height [mAU]	Area %
1	0.849	1	MM T	76.39623	15.23249	0.0958
2	8.587	1	MM T	7.96877e4	1970.01624	99.9042
Totals :				7.97641e4	1985.24872	

Figure S3. HPLC chromatogram of peptide **FTP**.

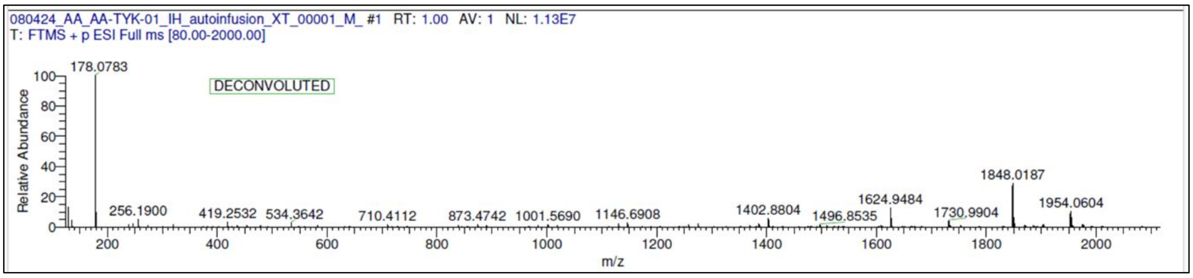

Figure S4. ESI-MS spectrum of peptide **FTP**.

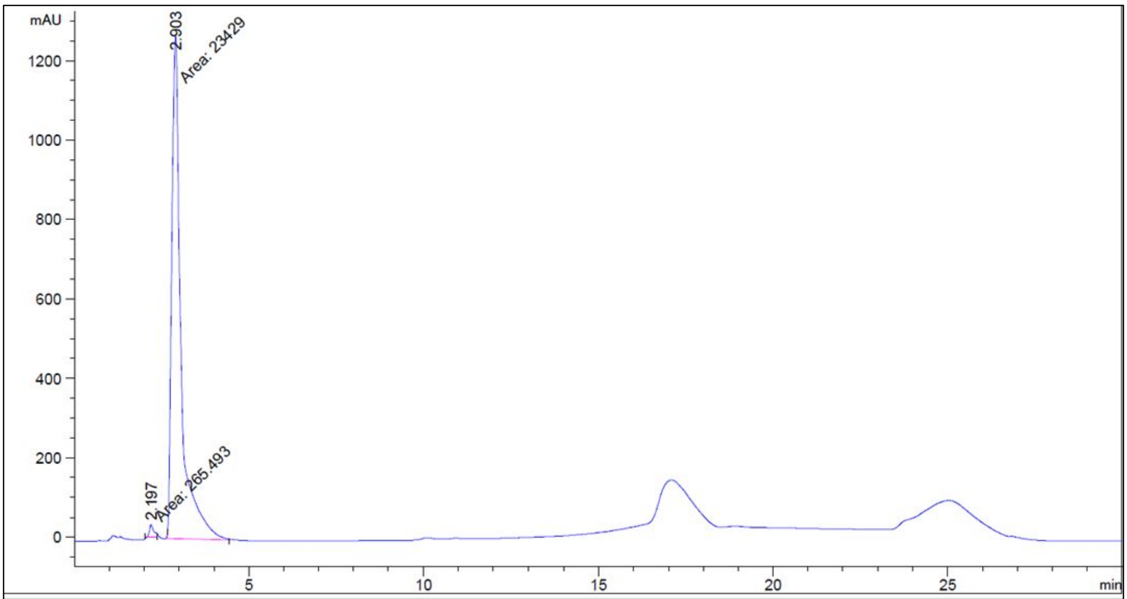

Peak #	RetTime [min]	Sig	Type	Area [mAU*s]	Height [mAU]	Area %
1	2.197	1	MM T	265.49255	31.10846	1.1205
2	2.903	1	MM T	2.34290e4	1265.42310	98.8795
Totals :				2.36945e4	1296.53156	

Figure S5. HPLC chromatogram of peptide **DTP**.

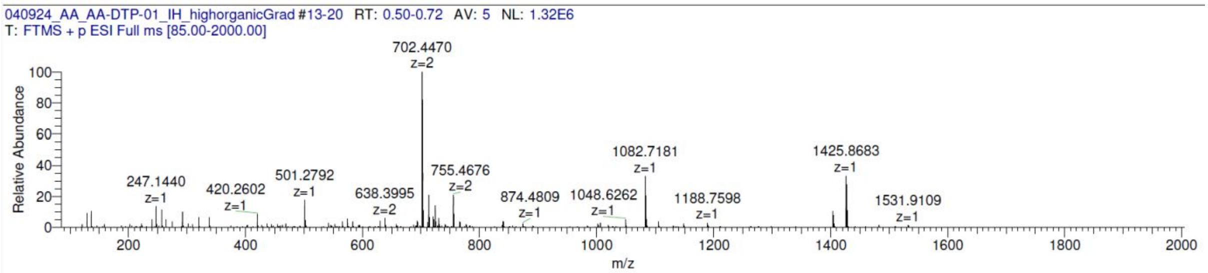

Figure S6. ESI-MS spectrum of peptide **DTP**.

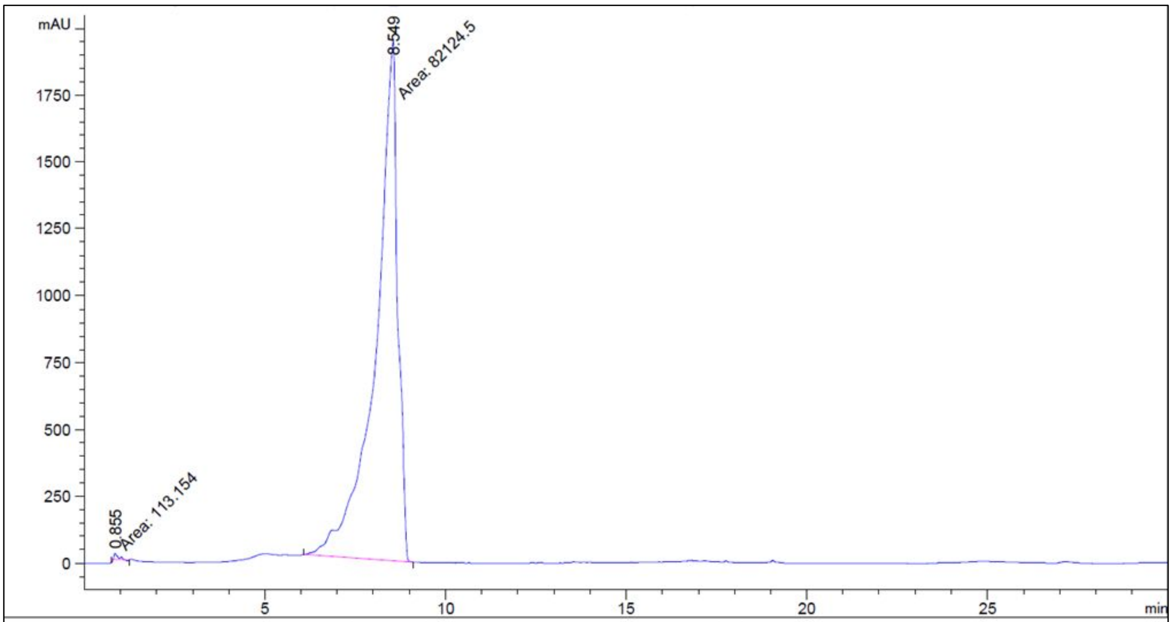

Peak #	RetTime [min]	Sig	Type	Area [mAU*s]	Height [mAU]	Area %
1	0.855	1	MM T	113.15363	21.49017	0.1376
2	8.549	1	MM T	8.21245e4	1942.87317	99.8624
Totals :				8.22376e4	1964.36334	

Figure S7. HPLC chromatogram of peptide **FDTP**.

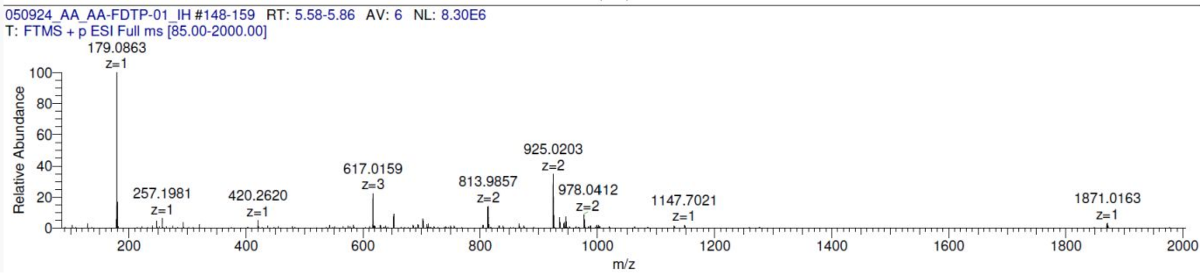

Figure S8. ESI-MS spectrum of **FDTP**.

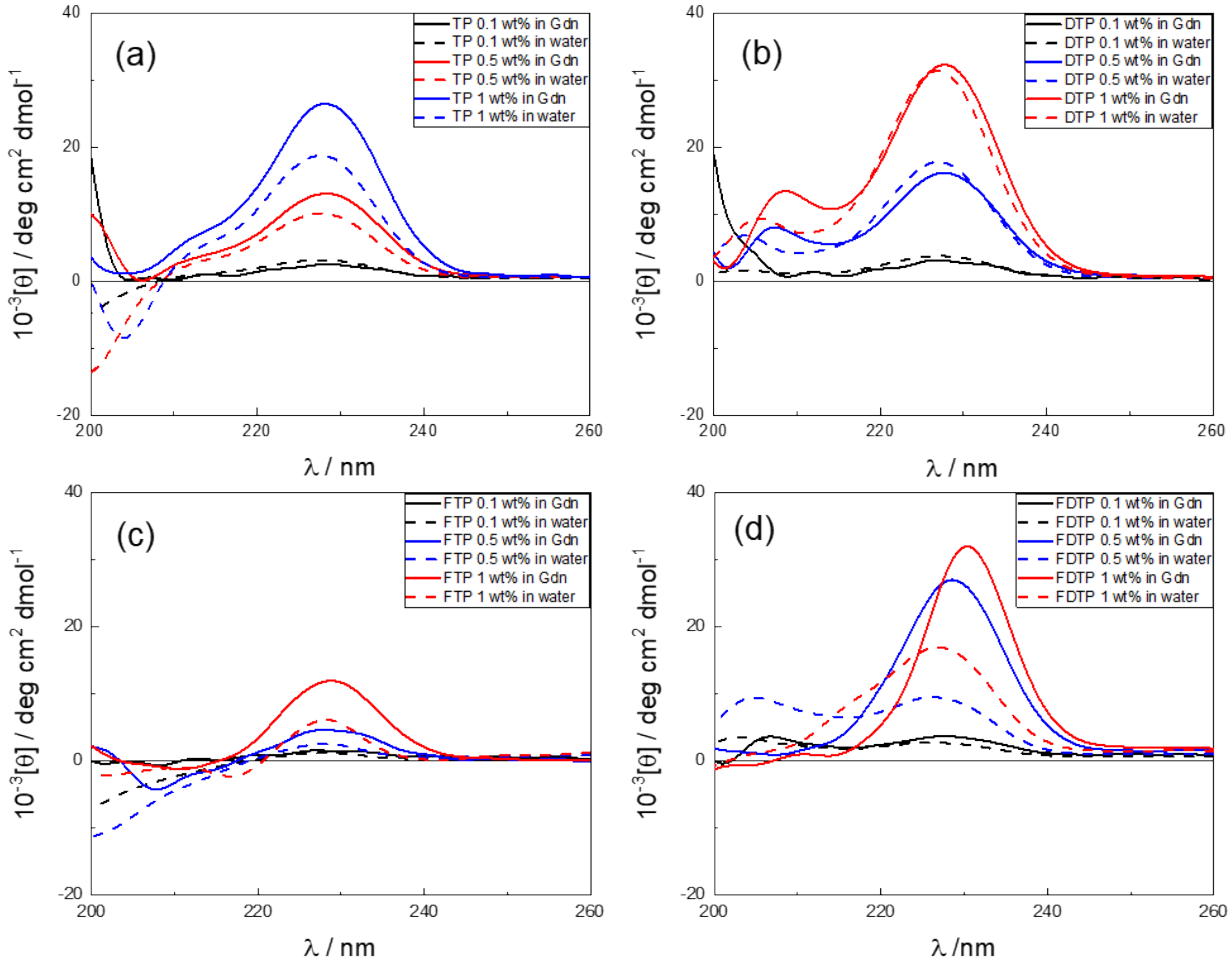

Figure S9. CD spectra for (a) **TP**, (b) **DTP**, (c) **FTP**, (d) **FDTP** at the concentrations shown and with and without 6M Gd.HCl.

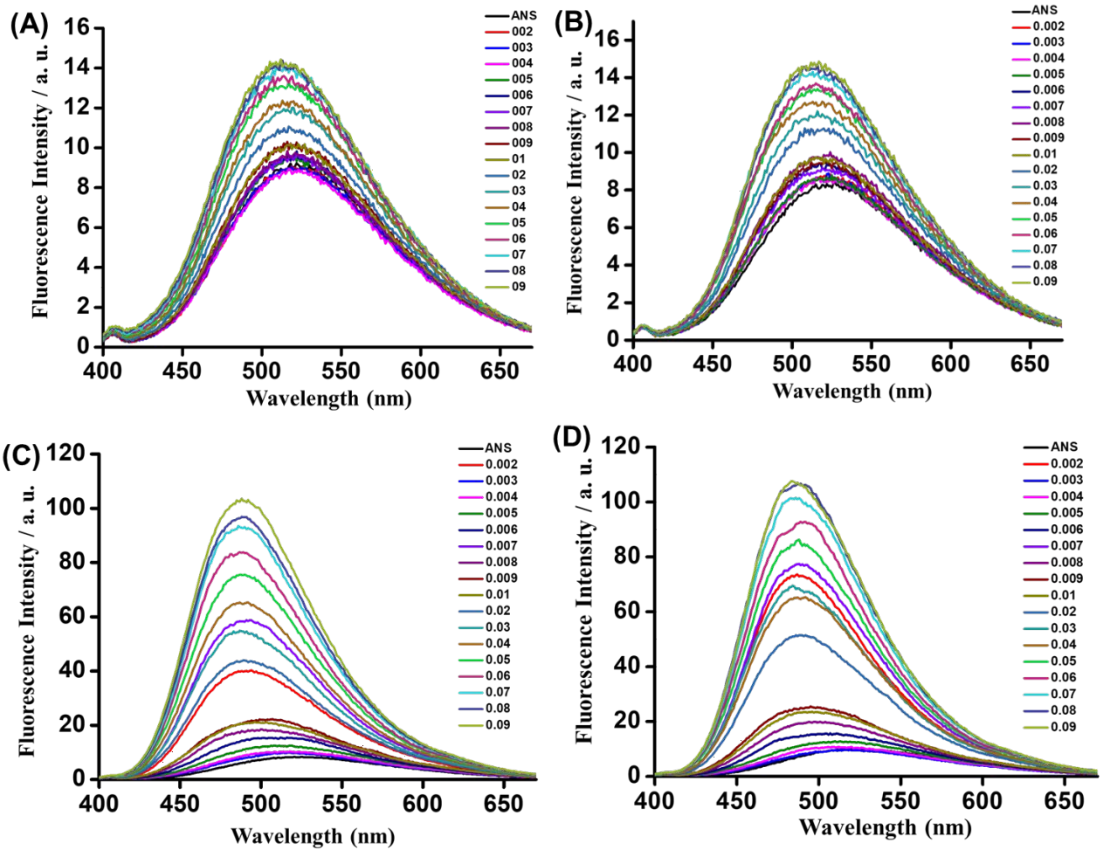

Figure S10. ANS fluorescence spectra at concentrations (wt%) shown for (A) **TP**, (B) **DTP**, (C) **FTP**, (D), and **FDTP**.
